# PathExpSurv: pathway expansion for explainable survival analysis and disease gene discovery

**DOI:** 10.1186/s12859-023-05535-2

**Published:** 2023-11-15

**Authors:** Zhichao Hou, Jiacheng Leng, Jiating Yu, Zheng Xia, Ling-Yun Wu

**Affiliations:** 1 IAM, MADIS, NCMIS, Academy of Mathematics and Systems Science, Chinese Academy of Sciences, Beijing, China; 2 School of Mathematical Sciences, University of Chinese Academy of Sciences, Beijing, China; 3 Computational Biology Program, Oregon Health & Science University, Portland, USA; 4 Department of Biomedical Engineering, Oregon Health & Science University, Portland, USA

**Keywords:** Survival analysis, Neural nerworks, Model interpretability, Pathways, Disease genes

## Abstract

**Background:**

In the field of biology and medicine, the interpretability and accuracy are both important when designing predictive models. The interpretability of many machine learning models such as neural networks is still a challenge. Recently, many researchers utilized prior information such as biological pathways to develop neural networks-based methods, so as to provide some insights and interpretability for the models. However, the prior biological knowledge may be incomplete and there still exists some unknown information to be explored.

**Results:**

We proposed a novel method, named PathExpSurv, to gain an insight into the black-box model of neural network for cancer survival analysis. We demonstrated that PathExpSurv could not only incorporate the known prior information into the model, but also explore the unknown possible expansion to the existing pathways. We performed downstream analyses based on the expanded pathways and successfully identified some key genes associated with the diseases and original pathways.

**Conclusions:**

Our proposed PathExpSurv is a novel, effective and interpretable method for survival analysis. It has great utility and value in medical diagnosis and offers a promising framework for biological research.

**Supplementary Information:**

The online version contains supplementary material available at 10.1186/s12859-023-05535-2.

## Introduction

When developing a predictive model in the area of biology and medicine, it is significant to balance the trade-off between accuracy and interpretability. Simple models like linear regression usually have high interpretability but don’t perform well, whereas the complex models based on deep learning can achieve good performance but it is hard to explain the black-box inside these models.

In this study, we investigated the accuracy and interpretability of survival models, which is specifically developed for dealing with censored data. Survival models are applied to perform time-to-event analysis in order to understand the relationships between the patients’ covariates and the risk of the event. The Cox proportional hazards model (CPH) [1], a semi-parametric regression model, was widely used in survival analysis. This model assumes that the log-risk of failure is a linear combination of the patient’s features. Although linear model has good interpretability, it might be too simplistic to just assume that the log-risk function is linear.

With the advent of machine learning, biomedical researchers were able to fit survival data with more sophisticated nonlinear log-risk functions [2–5]. Among these models, Faraggi and Simon [2] firstly incorporated the feed-forward neural network into the CPH, but this model with only a single hidden layer hadn’t showed great improvements beyond the CPH. DeepSurv [3] was an extension to Simon-Farragi’s network and configurable with multiple hidden layers. It employed a more complex deep neural network to model the relationships between the observed features and the patients’ risk of failure and showed improvements on the CPH when modeling the non-linear data. These neural network-based methods have high predictive performance, but they only leverage the fully connected neural networks, which maybe arbitrarily over-parameterized and lack of interpretability.

In order to design a biologically informed and sparse neural network, DeepOmix [6] utilized signaling pathways as the functional modules based on KEGG and Reactome databases to construct pathway-associated sparse network. Each node encoded some biological entity and each edge represented a known relationship between the corresponding entities. However, this model only considered the known and fixed functional modules in databases to design a sparse network, which might leave out some important factors. In fact, despite painstaking and manual curation, signaling pathways stored in databases still remained incomplete [7].

Therefore, it is necessary to make an exploration on the unknown space out of the prior information and identify some significant genes which may complement the original functional modules. In this paper, we presented PathExpSurv, a novel survival analysis method by exploiting and expanding the existing pathways. We firstly incorporated prior biological knowledge of signaling pathways into the neural network for survival analysis. In order to explore the possible unknown pathways with better performance, we further added the genes beyond the databases into the neural network pre-trained using the existing pathways, and continued to train a regularized survival analysis model, with a $L_1$ penalty that guarantees the sparse structure in the expanded pathways. By simultaneously exploiting the existing pathways and exploring the unknown pathways, PathExpSurv can gain an insight into the black-box model of neural network for survival analysis. We also performed several downstream analyses based on the expanded pathways and successfully identified some key genes associated with the diseases and original pathways.

## Methods

### Basic architecture

**Fig. 1:**
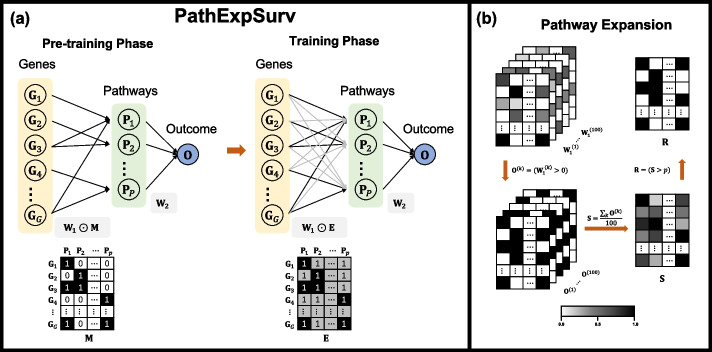
**a** Schematic overview of PathExpSurv. The basic architecture of the neural network consists 3 layers (gene layer, pathway layer and output layer). The connection between the gene layer and the pathway layer is determined by the pathway mask matrix, in which number 1 (black) means a non-penalized link representing a fixed relationship between gene and pathway in prior information, number 1 (grey) means a penalized link representing a possible relationship to be explored, and number 0 (white) means no link. The training scheme of PathExpSurv includes two phases, namely pre-training phase and training phase. In the pre-training phase, the prior pathway mask (M) is used to pre-train the model to achieve a relatively high and stable performance. In the training phase, a specific fully connected mask ($\textbf{E}$) with prior links and $L_1$-penalized non-prior links is used to train the model to explore the unknown space and obtain the expanded pathways. **b** Pipeline of pathway expansion. We first randomly chose 90% samples from the dataset to train the PathExpSurv model, and repeated 100 times to obtain the weight matrices between the gene layer and the pathway layer $\textbf{W}_1^{(k)}$ $(k=1,\ldots ,100)$. Then we transformed these matrices into binary matrices $\textbf{O}^{(k)}$ $(k=1,\ldots ,100)$, and calculated the occurrence probability matrix $\textbf{S}$ based on these binary matrices. Finally we obtained the expanded pathways matrix $\textbf{R}$ by filtering out the gene-pathway pairs with small occurrence probabilities

Suppose *G* is the number of genes, and *N* is the number of samples (patients). PathExpSurv uses a biologically informed neural network $f_\textbf{W}(\textbf{x})$ to predict the effects of a patient’s covariates on their hazard rate, with the input of gene expression $\textbf{x}\in \mathbb {R}^{1\times G}$ and the learnable weights $\textbf{W}$. Our main objective is to optimize the mean negative log partial likelihood:

$$\begin{aligned} l(\textbf{W}) = -\sum _{i=1}^{N} \delta _{i} \left[ f_\textbf{W}(\textbf{x}_{i}) -\log \left( \sum _{j:T_j\ge T_i} \exp \left( f_\textbf{W}(\textbf{x}_{j})\right) \right) \right] \end{aligned}$$

where $\delta _i\in \{0,1\}$ is the event indicator of *i*-th sample, $\textbf{x}_{i}\in \mathbb {R}^{1\times G}$ is the feature vector, and $T_i\in \mathbb {R}$ is the event time.

The basic architecture of neural network $f_\textbf{W}(\textbf{x})$ consists of 3 layers (Fig. 1a). The first layer is gene layer, the second layer is pathway layer and the third layer is the output layer. The nodes of first and second layers encode the genes and pathways respectively, and each edge represents the relationship between a gene and a pathway. The connections between the corresponding entities follow the pathway database such as KEGG and are encoded by a mask matrix $\textbf{M}$. We assume that the genes belonging to the same pathway have similar functions, so we constrain the weight $\textbf{W}_1$ between the gene and pathway layer to be non-negative. The output of neural network is calculated as:

$$\begin{aligned} f_\textbf{W}(\textbf{x},\textbf{M}) = \sigma \left( \sigma \left( \textbf{x} \cdot \left[ \textbf{W}_1 \odot \textbf{M} \right] \right) \cdot \textbf{W}_2 \right) \end{aligned}$$

where $\odot$ is the element-wise multiplication of two matrices, $\textbf{x}\in \mathbb {R}^{1\times G},\textbf{W}_1\in \mathbb {R}^{G\times P}_{+}, \textbf{M}\in \{0,1\}^{G\times P}, \textbf{W}_2\in \mathbb {R}^{P\times 1},\sigma =\text {tanh}$, and *P* is the number of pathways explored in the model.

### Two-phase training scheme

We proposed a novel optimization scheme consisting 2 phases (Fig. 1a): pre-training phase and training phase, in order to improve the performance of neural network by expanding the prior pathways.

During the pre-training phase, we utilized the prior pathways from the KEGG database to pre-train the model. We added a standard deviation term to the loss function due to the assumption that the genes in the prior functional modules are almost equally important. Then the objective function of pre-train phase became:

$$\begin{aligned} l_1(\textbf{W}) = -\sum _{i=1}^{n} \delta _{i} \left[ f_\textbf{W} \left( \textbf{x}_{i}, \textbf{M} \right) -\log \left( \sum _{j:T_j\ge T_i} \exp \left( f_\textbf{W} \left( \textbf{x}_{j}, \textbf{M} \right) \right) \right) \right] +\lambda \; \textbf{Std}\left( \textbf{W}_1 \odot \textbf{M} \right) \end{aligned}$$

where $\textbf{M}$ is the prior pathway mask matrix obtained from the KEGG database.

During the training phase, we changed the connections between the gene layer and the pathway layer to fully connected, and added a $L_1$ regularization term in order to select a few important genes from the genes outside the prior pathways. That is, we optimized the following loss:

$$\begin{aligned} l_2(\textbf{W})= -\sum _{i=1}^{n} \delta _{i} \left[ f_\textbf{W} \left( \textbf{x}_{i}, \textbf{E} \right) -\log \left( \sum _{j:T_j\ge T_i} \exp \left( f_\textbf{W} \left( \textbf{x}_{j}, \textbf{E} \right) \right) \right) \right] +\mu \left\| \textbf{W}_1 \odot \left( \textbf{1} - \textbf{M} \right) \right\| _1 \end{aligned}$$

where $\textbf{E} \in \{1\}^{G\times P}$ is the matrix of which the elements are all 1.

### Evaluation metric

When evaluating the performance of survival analysis, we need to consider the censored data. The concordance index (C-index) [8] is the most widely used evaluation metric in survival analysis. C-index is defined as:

$$\begin{aligned} {\textbf {C-index}} = \frac{\sum _{i, j} 1_{T_{j}<T_{i}} \cdot 1_{r(\textbf{x}_j)>r(\textbf{x}_i)} \cdot \delta _{j}}{\sum _{i, j} 1_{T_{j}<T_{i}} \cdot \delta _{j}} \end{aligned}$$

C-index expresses the proportion of concordant pairs in the dataset which estimates the probability that, for a random pair of individuals, the ordering of the predicted hazard risk of the two individuals is concordant with that of their true survival time.

### Pathway expansion

In order to identify the reliable genes complement to the prior pathways, we performed the following pathway expansion procedure as shown in Fig. 1b. Firstly, we selected 90% samples randomly from the dataset each time to train the PathExpSurv model. In this way, we repeated 100 times and obtained 100 different weight matrices between the gene layer and the pathway layer, $\textbf{W}_1^{(k)}$, $k=1,...,100$. Then we calculated the corresponding occurrence matrix as follows:

$$\begin{aligned} \textbf{O}^{(k)}(i,j) = \left\{ \begin{aligned} 1,&\quad \textbf{W}_1^{(k)}(i,j) > 0 \\ 0,&\quad \textbf{W}_1^{(k)}(i,j) = 0 \\ \end{aligned} \right. \end{aligned}$$

where $$k=1,...,100$$, $$i=1,...,G$$, $$j=1,...,P.$$

Secondly, we defined the occurrence probability of *i*-th gene in the *j*-th pathway as:

$$\begin{aligned} \textbf{S}(i,j) = \frac{\sum _{k=1}^{100}\textbf{O}^{(k)}(i,j)}{100} \end{aligned}$$

Finally, we sorted all the values in the occurrence probability matrix $\textbf{S}$ from biggest to smallest, and denoted the *n*-th biggest value as $p_{n}$. We extracted the top $\alpha K$ genes with highest occurrence probabilities to expand the prior pathways, where $\alpha$ is the parameter to control the size of expanded pathways and *K* is the total number of genes in the original pathways. The expanded pathways can be represented by the following incidence matrix:

$$\begin{aligned} \textbf{R}(i,j)=\left\{ \begin{aligned} 1,&\quad \textbf{S}(i,j) \ge p_{\lfloor (1+\alpha ) K+\frac{1}{2} \rfloor }\\ 0,&\quad \textbf{S}(i,j) < p_{\lfloor (1+\alpha ) K+\frac{1}{2} \rfloor }\\ \end{aligned} \right. \end{aligned}$$

## Results

### Data acquisition and experimental settings

To conduct computational experiments, we obtained 3 different survival datasets from UCSC Xena (https://xenabrowser.net/datapages/ ): (1) Breast Cancer Dataset (BRCA), (2) Lower Grade Glioma Dataset (LGG) and (3) Thyroid Cancer Dataset (THCA). For each cancer, we took the signaling pathways associated with the corresponding disease from KEGG DISEASE Database (https://www.kegg.jp/kegg/disease/ ) as the source of prior pathways, i.e. the functional modules. We only used gene expression data as the feature and the total number of genes in the original datasets is 60489. We did some preprocessing on the gene expression data. First, we transformed the read counts through $\log _2(x+1)$. Second, we selected the top variable genes of which the standard deviations among the patients were larger than 1. In this way, there were only 2005 (BRCA), 1061 (THCA) and 1126 (LGG) genes left. Third, we normalized the data into a standard normal distribution in order to overcome some problems like gradient vanishing in the neural network models. The detail information of cancer datasets and prior pathways were summarized in Additional file 1: Tables S1 and S2.

**Fig. 2:**
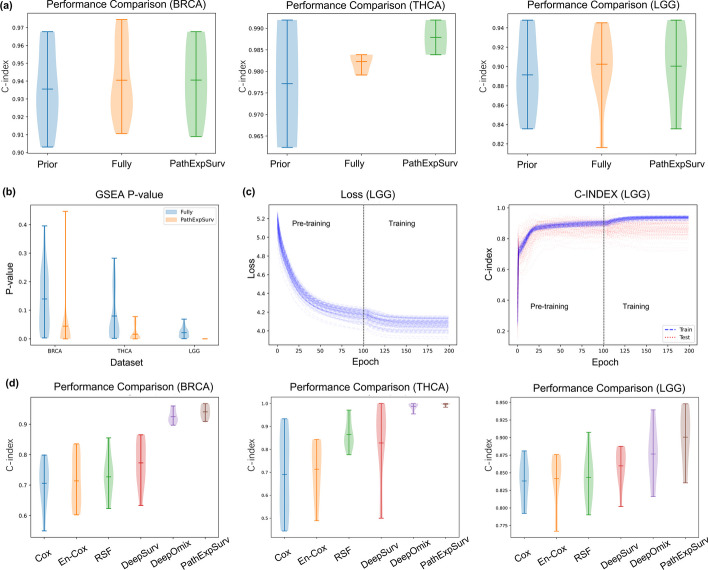
**a** Performance comparison on Prior Net, Fully-connected Net and PathExpSurv. Generally, the Fully-connected Net and PathExpSurv outperformed the Prior Net. On the THCA dataset, PathExpSurv even showed better result than the Fully-connected Net which had more learnable parameters. **b** GSEA p-values of the ranked genes list for each pathway. The GSEA p-values of PathExpSurv are significantly smaller than those of Fully-connected Net, indicating PathExpSurv has the ability to obtain meaningful expanded pathways and the results is more interpretable. **c** Example of training curves of the two-phase training. The loss and C-index showed significant improvement in the training phase. **d** Performance comparison on several methods of cancer survival analysis. The C-index results of 6 methods (Cox regression, Elastic-Net Cox model, Random Survival Forest, DeepSurv, DeepOmix and PathExpSurv) are shown, and PathExpSurv had best performance among these methods

Ten-fold cross-validation was used in the two-phase training. That is, we randomly divided the samples into training set and the testing set with the ratio of 9:1. We calculated the objective function, i.e., the loss function in the training set, and simultaneously computed the evaluation metric, i.e., C-index, to monitor the performance of models in both the training set and the testing set, as shown in Fig. 2c. The penalty weight $\lambda =1$ in the pre-training phase and $\mu =1$ in the training phase. We adopted the Adam optimizer to train our model, in which the learning rate was set to 0.05. The number of total epochs was 200 (i.e., 100 epochs for pre-training phase and 100 epochs for training phase), and the full batch was used. The parameter of pathway expansion $\alpha$ is set to 0.2 in this study.

### Performance of survival analysis

We first compared the performance of PathExpSurv with two baseline models: Prior Net, Fully-connected Net. The Prior Net model utilized the sparse neural network derived from the prior pathways, and was trained using the same loss in the pre-training phase of PathExpSurv, which included a standard deviation penalty. On the other hand, the Fully-connected Net model employed the fully connected neural network, and was trained through the loss with the $L_1$ penalty in the training phase of PathExpSurv. For fair comparison, the number of epochs of the training process was set to 200 for both Prior Net and Fully-connected Net. The training scheme of PathExpSurv can be regarded as a mixture of two baseline models, comprising 100 epochs of pre-training with Prior Net, followed by another 100 epochs of training with Fully-connected Net. We performed 10-fold cross validation and the results were showed in Fig. 2a. As expected, the Fully-connected Net and PathExpSurv outperformed the Prior Net. On the THCA dataset, PathExpSurv even showed better result than the Fully-connected Net which had more learnable parameters.

We further investigated and compared the interpretability of PathExpSurv with the Fully-connected Net. We extracted the ranked gene list for each pathway from the weight matrix $\textbf{W}_1$, and performed Gene Set Enrichment Analysis (GSEA) to test whether the ranked gene list is closely associated with some functional term. The p-values of the top enriched term for each pathway were shown in Fig. 2b. The GSEA p-values of PathExpSurv were significantly smaller than those of Fully-connected Net, indicating that PathExpSurv had the tendency to discover some genes which were closely related with each other and was more explainable than Fully-connected Net. Together with the results in Fig. 2a, we can conclude that the Prior Net has good interpretability but its performance might be limited, while the Fully-connected Net has higher performance but its interpretability might be poor. And our PathExpSurv could balance the performance and the interpretability well.

For accurately evaluating the roles of pre-training phase and training phase, we performed two-phase training scheme for 100 random experiments and computed the means and standard deviations of the results. Table 1 displayed the results of these two phases. Fig. 2c showed the training curve on LGG, and the training curves of other datasets were shown in Additional file 1: Fig. S1. We found that the optimal C-indices of training phases were mostly better than those of pre-training phases, which meant that the training of pre-trained networks learned more useful information beyond the prior pathway modules.

**Table 1:** Means and standard deviations of C-index in pre-training and training phase

Dataset	Samples	Pre-training phase	Training phase
BRCA	Training set	93.66 ± 0.42	**95.61** ± 0.39
	Testing set	92.81 ± 2.13	**93.02** ± 1.93
THCA	Training set	98.64 ± 0.31	**98.88** ± 0.30
	Testing set	98.48 ± 3.25	**98.99** ± 1.41
LGG	Training set	90.17 ± 1.05	**93.69** ± 0.78
	Testing set	**88.60** ± 3.61	88.34 ± 3.78

The best C-index in pre-training phase and training phase is marked in bold

Finally, to evaluate the performance of PathExpSurv against state-of-the-art methods, we conducted 10-fold cross validation and compared the final C-index values in the testing set for each method. The performance of PathExpSurv was compared with five typical survival analysis methods: the Cox proportional hazards model [1], Elastic-Net Cox model (En-Cox), Random Survival Forest (RSF) [9], DeepSurv [3], and DeepOmix [6]. As shown in Fig. 2d, PathExpSurv had best performance among these methods. In general, neural networks-based models (DeepSurv, DeepOmix and PathExpSurv) are superior to other methods (Cox, En-Cox and RSF). It is worthy to note that, the poor performance of DeepSurv is partially attributed to the over-fitting in the training dataset, while the prior information utilized in PathExpSurv and DeepOmix can help them to avoid the over-fitting.

### Gene selection and pathway expansion

**Fig. 3:**
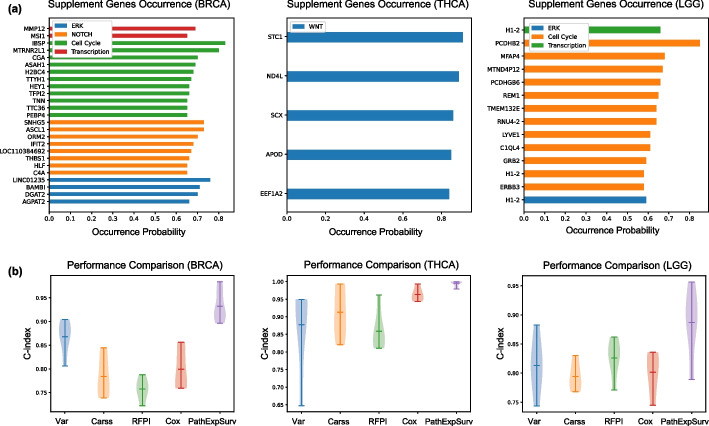
**a** Occurrence probability of the supplement genes. **b** Performance comparison on several gene selection methods. PathExpSurv showed best performance compared to other 4 methods (Var, Carss, RFPI and Cox)

Applying the pathway expansion procedure, we identified the supplement genes of each prior pathway for each dataset, as shown in Table 2. In each disease dataset, the number of supplement genes is 20% of the total size of the original pathways. The occurrence probabilities of these supplement genes were exhibited in Fig. 3a, most of which are larger than 0.6, indicating these genes could be reliably identified. On the one hand, these supplement genes were significantly related to the corresponding pathway, as validated by the enrichment analysis and the recoverability testing in this section. On the other hand, these supplement genes were also closely associated with the corresponding disease, which would be demonstrated in next section.

**Table 2:** List of prior pathways and supplement genes

Dataset	Pathway	Original	Expanded	Supplement genes
BRCA	ERK signaling	18	22	*AGPAT2, BAMBI, DGAT2, LINC01235*
	PI3K signaling	15	15	–
	WNT signaling	46	46	–
	NOTCH signaling	14	22	*LOC110384692, C4A, HLF, SNHG5, ASCL1, ORM2, IFIT2, THBS1*
	Nuclear receptor signaling	5	5	–
	Cell cycle	6	17	*IBSP, HEY1, TNN, H2BC4, MTRNR2L1, CGA, TFPI2, TTYH1, ASAH1, PEBP4, TTC36*
	Transcription	9	11	*MMP12, MSI1*
THCA	ERK signaling	12	12	–
	WNT signaling	5	10	*STC1, APOD, EEF1A2, ND4L, SCX*
	Transcription	11	11	–
LGG	ERK signaling	19	20	*H1-2*
	PI3K signaling	13	13	–
	Calcium signaling	15	15	–
	Cell cycle	13	25	*REM1, C1QL4, MTND4P12, GRB2, RNU4-2, LYVE1, TMEM132E, PCDHB2, ERBB3, H1-2, PCDHGB6, MFAP4*
	Transcription	9	10	*H1-2*

We compared PathExpSurv with several other gene selection methods, including three filter methods summarized in [10]: variance filter (Var), Carss filter (Carss), and random forest permutation importance filter (RFPI). Additionally, we utilized the Cox proportional hazards model [1] to select genes with high absolute weight values, which we referred to as the Cox score method (Cox). The filtered gene set size was set to be the number of genes of all the expanded pathways. Subsequently, we inputted the filtered genes into fully-connected networks for survival analysis. The results presented in Fig. 3b showed that PathExpSurv significantly outperformed other gene selection methods.

**Fig. 4:**
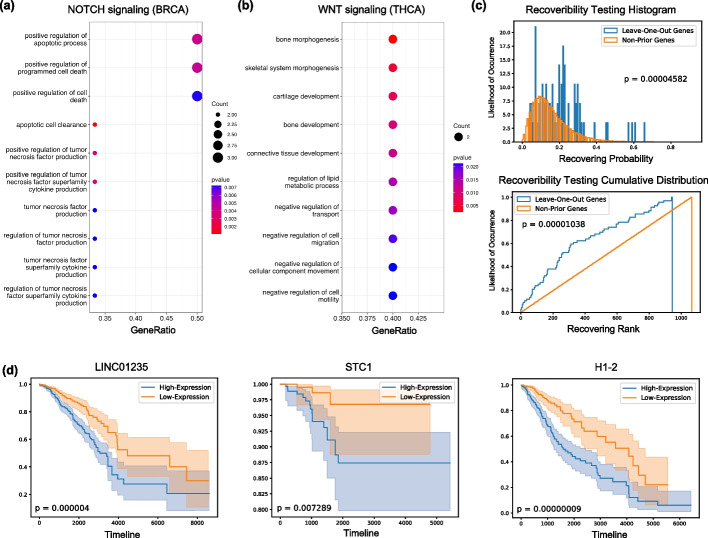
**a** GO term enrichment analysis result of the supplement genes of NOTCH signaling pathway for BRCA, and **b** WNT signaling pathway for THCA. **c** Comparison of the recovering probability (top) and rank (bottom) distributions of leave-one-out genes and non-prior genes. The p-values of Kolmogorov–Smirnov test are shown in the figure. **d** Kaplan–Meier curves of single-gene survival analysis for three most significantly different genes ($p < 0.05$)

We then performed Gene Ontology (GO) term enrichment analysis on the supplement genes of each pathway, so as to discover the relationships between original pathway and expanded pathway. As shown in Additional file 1: Fig. S3 and Table S5, the supplement genes of ERK signaling pathway for BRCA are enriched in *glycerolipid biosynthetic process* ($p=0.000720304$) and *glycerolipid metabolic process* ($p=0.002490982$), which are closely related to ERK signaling [11]. The supplement genes of NOTCH signaling pathway for BRCA are enriched in *positive regulation of tumor necrosis factor production* ($p=0.003496794$) and *positive regulation of tumor necrosis factor superfamily cytokine production* ($p=0.003496794$), as shown in Fig. 4a and Additional file 1: Table S6. Fernandez et al. [12] showed that tumor necrosis factor-$\alpha$ modulate NOTCH signaling in the bone marrow microenvironment during inflammation. The supplement genes of WNT signaling pathway for THCA are enriched in *bone morphogenesis* ($p=0.00252103$) and *skeletal system morphogenesis* ($p=0.005187936$), as shown in Fig. 4b and Additional file 1: Table S7. WNT signaling activates bone morphogenetic protein 2 expression [13].

We also conducted a simulation experiment, named recoverability testing, to test whether PathExpSurv could recover the meaningful genes closely related to the prior pathway. We adopted the leave-one-out cross-validation strategy for this experiment. In this experiment, we removed one gene from the prior pathway at a time and applied PathExpSurv 100 times to check how many times the leave-one-out gene can be recovered. We then compared the recovering probabilities of leave-one-out genes and non-prior genes. The two-sample Kolmogorov-Smirnov test reveals that there is a significant difference between the recovering probability (rank) distributions of leave-one-out genes and non-prior genes (Fig. 4c). The discrepancy of the two distributions showed that the leave-one-out genes were more likely to be recovered, which might indicate that PathExpSurv had the ability to identify the genes significantly related to the corresponding pathway.

### Disease gene discovery

The supplement genes were identified because they could enhance the performance of survival analysis, implying their close association with the corresponding disease. We conducted a literature search and discovered some promising evidence to support this notion. These genes could be further investigated and potentially used as the additional important indicators for the disease.

For breast cancer, Wang et al. [14] showed the close relationship between the expression of *BAMBI* and the proliferation and migration of breast cancer. The high expression of *LINC01235* was associated with poor prognosis of breast cancer patients [15]. *IFIT2* was considered a tumor suppressor in breast cancer [16], as it had been identified to inhibit cancer cell growth and migration, and promoted cell apoptosis. Chi et al. [17] demonstrated that small nucleolar RNA host gene 5 (*SNHG5*) promoted breast cancer cell proliferation both in vitro and in vivo. HLF regulates ferroptosis, development and chemoresistance of triple-negative breast cancer by activating tumor cell-macrophage crosstalk [18]. The expression of *THBS1* in breast cancer was associated with poor metastasis-free survival [19]. Knockdown of *PEBP4* inhibited breast cancer cell proliferation in vitro and tumor growth in vivo [20]. The abnormal expression of the *IBSP* gene was closely related to bone metastasis, increased malignant risk and the poor prognosis of breast cancer [21]. *TFPI2* was down-regulated in breast cancer tissues and cell lines, and was associated with poor prognosis of patients diagnosed with breast cancer [22]. Zhou et al. [23] found that increased *CGA* expression was significantly associated with a poor prognosis in patients with breast cancer. *H2BC4* was overexpressed in breast cancer [24]. *MSI1* was a negative prognostic indicator of breast cancer patient survival, and was indicative of tumor cells with stem cell-like characteristics [25].

For thyroid cancer, Hayase et al. [26] demonstrated that *STC1* was highly expressed in thyroid tumor cell line and thyroid tumor tissues. The expression level of *APOD* showed significant differences in the high- and low-risk groups of differentiated thyroid cancer recurrence [27]. *EEF1A2* was previously suggested as driver of tumor progression and potential biomarker [28].

For lower grade glioma, *ERBB3* showed marked underexpression in most glioblastomas [29]. *GRB2* was largely involved in multiple tumor malignancies [30]. Yang et al. [31] indicated that *MFAP4* could be used as novel biomarker for developing therapies against human cancers.

We also performed the single-gene survival analysis to validate the significance of the newly-found disease genes. For one specific gene, we divided the dataset into two groups: high expression group contained the top 50% gene expression level and low expression group contained the others. Then we ploted the Kaplan-Meier curves of the two groups, and identified the most significantly different genes ($p<0.05$). We displayed three examples (*LINC01235, STC1, H1-2*) in Fig. 4d, while the complete curves of all the significant genes were shown in Additional file 1: Fig. S4. For BRCA, we identified key genes: *LINC01235*, *TTC36*, *H2BC4*, *THBS1*, *AGPAT2*, *MMP12*. For THCA, we got *STC1*, *ND4L*, *APOD*. For LGG, we obtained *H1-2*, *LYVE1*, *MFAP4*, *PCDHGB6*. These genes were differentially expressed between two groups and might contribute to the performance improvement of PathExpSurv.

## Limitations and discussion

The supplement genes identified by PathExpSurv are useful since they can be interpreted as the unknown important genes to complement the original pathways. First, the expanded pathways can be used to enhance the predictive performance of many bioinformatics models based on pathways, as in our comprehensive survival analysis experiments. Second, the supplement genes are important for diagnosing and studying related diseases. Compared with the single gene identified by other bioinformatics methods such as differential expressed gene analysis, the supplement genes identified by PathExpSurv are associated with specific pathways respectively, therefore can provide more insightful hypotheses for investigating the molecular mechanisms of diseases. Last but not least, the supplement genes are also helpful to reconstruct potentially incomplete pathways and fill the gap in the existing database.

However, it is worth noting that we need to be very cautious when interpreting the supplement genes identified by PathExpSurv. First, the supplement genes are identified through statistical analysis based on the mathematical model. The associations between the supplement genes and the respective pathways and diseases are purely inferred by computational algorithm, and are not guaranteed absolute truth. Whether the supplement genes belongs to the respective pathways and their concrete roles in the pathways require further validation. Therefore, the improved predictive performance after introducing the supplement genes is less interpretable than that using only the known genes in the pathways. The users should use the supplement genes carefully and avoid to provide misleading conclusions. Second, as most machine learning approaches, the supplement genes and their associations with the pathways and diseases are predicted based on the model learned from the training data, and the results on different datasets may be varied. The users should carefully select the training datasets according to the purpose and design of their experiments to obtain reliable and convincing results.

PathExpSurv offers a novel and effective method for better survival analysis with high interpretability. When implementing the method as a practical tool for clinicians, it is important to pay attention to the utilization of PathExpSurv’s advantages. First, the prior pathways are crucial input and should be carefully selected by the clinicians based on their knowledge of diseases and patients. Second, besides the survival risk scores predicted by the model, the tool should also output the expanded pathways with the supplement genes so that the clinicians can justify the results. Third, different datasets can be used in two phases in order to balance the performance and the computation time. For example, a large dataset from public databases is used in the pre-training phase to improve the reproducibility and reliability, while a small dataset from the targeting patients is used in the training phase to increase the sensitivity and specificity. Fourth, visualization and enrichment analysis of the expanded pathways and their relationships with diseases are necessary for understanding and interpreting the results.

Although PathExpSurv has achieved good performance and exhibited great explainability, there still exist some directions to improve this model. First, the current approach for selecting genes beyond the database is based on the LASSO method, and we can also consider some attribution methods such as DeepLIFT [32], DeepExplain [33] and LIME [34]. Second, PathExpSurv only employed a 3-layer neural network, and more sophisticated architecture might further improve the performance and interpretability. Third, the training scheme of PathExpSurv consisted of two phases, we can design a more complex training way to adjust the pathways step by step. Furthermore, PathExpSurv could be regarded as a high-level framework which might be applied to all kinds of prediction tasks.

## Conclusion

In this paper, we proposed a novel survival analysis method, named PathExpSurv, which exploited a two-phase training scheme to firstly pre-train the biologically informed neural network and then further train it to make an exploration beyond the prior pathway database. We showed that PathExpSurv can improve the performance of survival analysis while keep good interpretability of the model. Furthermore, our method can also obtain valuable supplement genes which are significantly associated with the prior pathways and the diseases.

## Supplementary information

Supplementary materials are available at BMC Bioinformatics online, including the foundation of survival time analysis, the detailed information of data, supplementary experiment results and downstream analysis of expanded pathways.

### Supplementary Information

**Additional file 1.** Supplementary materials.

## Data Availability

Raw data of are available in https://xenabrowser.net/datapages/. The datasets generated during the current study and code are all available in https://github.com/Wu-Lab/PathExpSurv/tree/main/Dataset.

## Code availability

Python source code of PathExpSurv is freely available at https://github.com/Wu-Lab/PathExpSurv.
