# Supplementary material for: PathExpSurv: pathway expansion for explainable survival analysis and disease gene discovery
Source: BMC Bioinformatics. 2023 Nov 15;24:434. doi: 10.1186/s12859-023-05535-2 (PMC10648621; doi:10.1186/s12859-023-05535-2)
Supplement: Supplementary file 1 — Additional file 1. Supplementary materials. [file 12859_2023_5535_MOESM1_ESM.pdf]

Supplementary Materials

PathExpSurv: Pathway Expansion for Explainable Survival Analysis and Disease Gene Discovery

Zhichao Hou [1,2], Jiacheng Leng [1,2], Jiating Yu [1,2], Zheng Xia [3,4,*] and Ling-Yun Wu [1,2,*]

[1]IAM, MADIS, NCMIS, Academy of Mathematics and Systems Science, Chinese Academy of Sciences, Beijing 100190, China, [2]School of Mathematical Sciences, University of Chinese Academy of Sciences, Beijing 100049, China, [3]Computational Biology Program, Oregon Health & Science University, Portland, OR 97239, USA, [4]Department of Molecular Microbiology and Immunology, Oregon Health & Science University, Portland, OR 97239, USA.

Contents

1 Foundation of Survival Time Analysis **2**
1.1 Notations of Common Terms in Survival Analysis 2
1.2 Cox Proportional Hazards Model 2

2 Details of Data **2**
2.1 Cancer Datasets 2
2.2 KEGG Signaling Pathways 3

3 Experiment Results **5**
3.1 Two-phase Training 5
3.2 Retraining 5

4 Analysis of Expanded Pathways **7**
4.1 Gene Ontology (GO) Term Enrichment Analysis 7
4.2 Single-Gene Survival Analysis 9

1 Foundation of Survival Time Analysis

1.1 Notations of Common Terms in Survival Analysis

Survival analysis is used to handle the survival data which is right-censored (i.e. some patients may leave the study or the study ends before an event occurs).

The compositions of survival data are: a patient's observed covariates $\mathbf{x}$, a survival time T, and an event indicator $\delta \in \{0, 1\}$.

The survival function is denoted as

$$S(t) = P(T > t),$$

which signifies the probability that the failure time is latter than t.

The hazard function is defined as:

$$h(t) = \lim_{\Delta t \to 0} \frac{P(t \leq T < t + \Delta t \mid T \geq t)}{\Delta t}$$

It is the probability an individual will not survive for an additional time Δt, given they have already survived up to time t.

1.2 Cox Proportional Hazards Model

The Cox proportional hazards model is a common method for modeling an individual's survival given the feature $\mathbf{x}$. The model assumes that the hazard function is composed of two non-negative functions: a baseline hazard function, $h_0(t)$, and a risk score, $r(\mathbf{x}) = \exp(\mathbf{x}^T\beta)$, defined as the effect of an individual's obeserved covariates on the baseline hazard. Then the hazard function is assumed to have the form:

$$h(t \mid x) = h_0(t) \exp\left(\mathbf{x}^T \beta\right)$$

The likelihood of the event to be observed occurring for subject i at time T_i can be written as:

$$L_i(\beta) = \frac{h(T_i \mid \mathbf{x}_i)}{\sum_{j:T_j \geq T_i} h(T_j \mid \mathbf{x}_j)} = \frac{h_0(T_i) \exp\left(\mathbf{x}_i^\top \beta\right)}{\sum_{j:T_j \geq T_i} h_0(T_i) \exp\left(\mathbf{x}_j^\top \beta\right)} = \frac{\exp\left(\mathbf{x}_i^\top \beta\right)}{\sum_{j:T_j \geq T_i} \exp\left(\mathbf{x}_j^\top \beta\right)}$$

The Cox log partial likelihood function is defined as:

$$L_c(\beta) = \log\left(\prod_{i:\delta_i=1} L_i(\beta)\right) = \sum_{i=1}^{n} \delta_i \left[\mathbf{x}_i^\top \beta - \log\left(\sum_{j:T_j \geq T_i} \exp\left(\mathbf{x}_j^\top \beta\right)\right)\right]$$

The commonly used metric in survival analysis is concordance index (C-index):

$$\textbf{C-index} = \frac{\sum_{i,j} 1_{T_j < T_i} \cdot 1_{r(\mathbf{x}_j) > r(\mathbf{x}_i)} \cdot \delta_j}{\sum_{i,j} 1_{T_j < T_i} \cdot \delta_j}$$

2 Details of Data

2.1 Cancer Datasets

For data acquisition, we obtained 3 different survival datasets from UCSC Xena: (1) Breast Cancer Dataset (BRCA), (2) Lower Grade Glioma Dataset (LGG), (3) Thyroid

Cancer Dataset (THCA). Table S1 shows the number of samples and selected genes of 3 different datasets. For each cancer, we only used gene expression data as the feature and the total number of genes was 60489. Then we did some preprocessing for the gene expression data. First, we transformed the read counts through $\log_2(x+1)$. Second, we selected the top variable genes of which the standard deviations among the patients were larger than 1. In this way there were only 2005 (BRCA), 1061 (THCA) and 1126 (LGG) genes left. Third, we normalized the data into a standard normal distribution in order to overcome some problems like gradient vanishing in the deep learning models.

Table S1: Datasets basic information.

	(num-samples, num-genes)
BRCA	(1194, 2005)
THCA	(567,1061)
LGG	(524,1126)

2.2 KEGG Signaling Pathways

We took the prior pathways as the functional modules and the source of the prior signaling pathways was KEGG DISEASE Database. The details of the composition of the pathways are showed in Table S2 .

Table S2: Compositions of the prior pathways.

Disease	Pathway	Composition
BRCA	ERK signaling	*HRAS/BRAF/EGFR/GRB2/KRAS/RAF1/MAP2K1/ MAP2K2/ERBB2/ARAF/EGF/FGFR1/FGFR2/MAPK1/ MAPK3/NRAS/SOS1/SOS2*
	PI3K signaling	*PTEN/EGFR/PIK3CA/PIK3CB/MTOR/PIK3CD/AKT1/AKT2/ AKT3/ERBB2/FGFR1/RPS6KB1/RPS6KB2/FGFR2/EGF*
	WNT signaling	*MYC/FZD1/FZD4/FZD7/WNT5B/FZD6/FZD8/FZD9/TCF7/ TCF7L2/FRAT1/FZD3/WNT1/WNT2/WNT3/WNT5A/WNT6/ WNT3A/WNT7A/WNT7B/WNT8A/WNT10B/WNT8B/WNT2B/ WNT9A/WNT9B/WNT16/DVL1/DVL2/DVL3/APC/WNT10A/ LRP6/LRP5/FZD10/CCND1/WNT4/CTNNB1/FZD5/ FZD2/LEF1/FRAT2/TCF7L1/GSK3B/AXIN1/AXIN2*
	NOTCH signaling	*DLL1/JAG2/HEY2/HEY1/DLL4/HES5/HEYL/ ERBB2/HES1/NOTCH1/FLT4/JAG1/NOTCH4/ DLL3*
	Nuclear receptor signaling	*MYC/NCOA1/NCOA3/CCND1/ESR1*
	Cell cycle	*RB1/E2F1/E2F2/E2F3/CCND1/CDK4*
	Transcription	*GADD45G/CDKN1A/BAK1/POLK/BAX/ GADD45B/DDB2/GADD45A/TP53*
THCA	ERK signaling	*NRAS/ARAF/BRAF/RAF1/MAP2K1/MAP2K2/ MAPK1/MAPK3/RET/HRAS/KRAS/CCND1*
	WNT signaling	*TCF7L1/TCF7L2/LEF1/MYC/CCND1*
	Transcription	*GADD45B/GADD45G/BAX/BAK1/DDB2/ POLK/PAX8/RXRA/RXRB/RXRG/PPARG*
LGG	ERK signaling	*KRAS/RAF1/NRAS/BRAF/PDGFA/PDGFB/EGFR/ PDGFRA/PDGFRB/HRAS/GRB2/CCND1/MAPK1/ MAPK3/MAP2K1/MAP2K2/ARAF/SOS1/SOS2*
	PI3K signaling	*PTEN/EGFR/PDGFRA/PIK3CA/PIK3CB/MTOR/ PIK3CD/AKT1/AKT2/AKT3/RPS6KB1/RPS6KB2/BAD*
	Calcium signaling	*CALM1/CAMK1/EGFR/CALM2/PDGFRA/CALM3/ CAMK4/CAMK2A/CAMK2B/CAMK2D/CAMK2G/ CAMK1G/PLCG1/PLCG2/CAMK1D*
	Cell cycle	*CCND3/MDM2/CDKN1A/CDKN2A/RB1/E2F1/E2F2/ E2F3/CCND1/TP53/CDK4/CDK6/CCND2*
	Transcription	*GADD45G/CDKN1A/BAK1/POLK/BAX/ GADD45B/DDB2/GADD45A/TP53*

3 Experiment Results

3.1 Two-phase Training

Table S3 displayed the exact results of two phases, and Figure S1 showed the training curves. We found that the optimal C-indices of training phase were mostly better than those of pre-training phase, which means that the training of pre-trained network learned more useful information beyond the prior pathways modules.

Table S3: Results of pre-training & training phase.

Disease	Item	Pre-training Phase	Training Phase
BRCA	Loss	4.76112 ± 0.03988	**4.69065** ± 0.04615
	C-index (train)	0.93660 ± 0.00425	**0.95611** ± 0.00385
	C-index (test)	0.92812 ± 0.02134	**0.93020** ± 0.01934
THCA	Loss	3.58619 ± 0.16515	**3.51818** ± 0.16522
	C-index (train)	0.98640 ± 0.00315	**0.98880** ± 0.00303
	C-index (test)	0.98481 ± 0.03253	**0.98994** ± 0.01414
LGG	Loss	4.16411 ± 0.05032	**4.07388** ± 0.05227
	C-index (train)	0.90174 ± 0.01049	**0.93691** ± 0.00783
	C-index (test)	**0.88602** ± 0.03614	0.88339 ± 0.03782

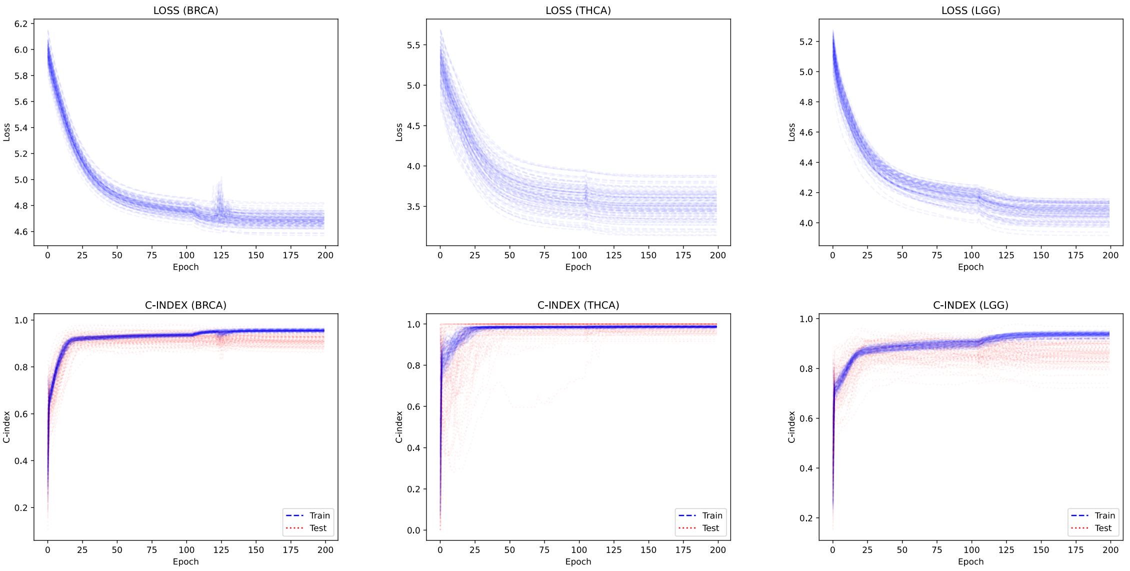

Figure S1: Training curves of pre-training and training phase. The model achieves better performance in training phase (2nd 100 epoches) than that in pre-training phase (1st 100 epoches).

3.2 Retraining

Table S4 and Figure S2 compared the results of model with original pathways mask and expanded pathways mask. The results showed that the expanded pathways achieved better performance than original pathways. We found that the expanded pathways outperformed

significantly original modules at epoch 5, which indicated that the newly learned meta-pathways could help predictor to learn more quickly. We also noticed that the C-index on the training set of expanded pathways was worse than that of original pathways, but the result was converse on testing set, which might demonstrate that the meta-pathways overcome the over-fitting problem in some way.

Table S4: Comparison of original pathways and expanded pathways.

Disease	Item	Original	Expanded
BRCA	Loss	4.76112 ± 0.03988	**4.78944** ± 0.03473
	C-index (Train Optimal)	**0.93660** ± 0.00425	0.92501 ± 0.00466
	C-index (Train Epoch 5)	0.73742 ± 0.01801	**0.78574** ± 0.03140
	C-index (Test Optimal)	0.92812 ± 0.02134	**0.92828** ± 0.02094
	C-index (Test Epoch 5)	0.71595 ± 0.07056	**0.77376** ± 0.06358
THCA	Loss	3.58619 ± 0.16515	**3.55375** ± 0.16606
	C-index (Train Optimal)	**0.98640** ± 0.00315	0.98594 ± 0.00396
	C-index (Train Epoch 5)	0.86103 ± 0.04687	**0.91480** ± 0.03014
	C-index (Test Optimal)	0.98481 ± 0.03253	**0.98906** ± 0.01905
	C-index (Test Epoch 5)	0.83347 ± 0.15593	**0.90224** ± 0.10201
LGG	Loss	4.16411 ± 0.05032	**4.14754** ± 0.04814
	C-index (Train Optimal)	0.90174 ± 0.01049	**0.90427** ± 0.00966
	C-index (Train Epoch 5)	0.74366 ± 0.02394	**0.75932** ± 0.02198
	C-index (Test Optimal)	**0.88602** ± 0.03614	0.87816 ± 0.04556
	C-index (Test Epoch 5)	0.74234 ± 0.06984	**0.75472** ± 0.06494

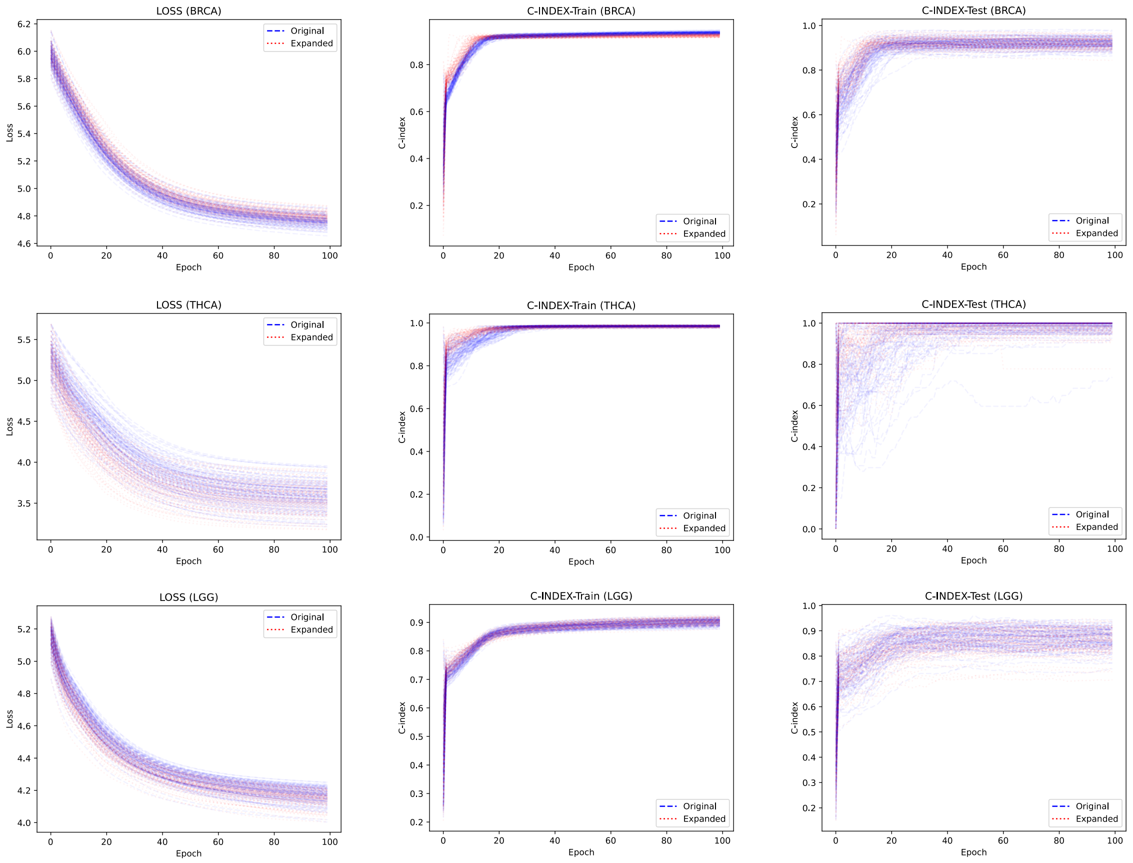

Figure S2: Training curves of retraining. The expanded-pathways model can achieve better performance quickly and finally outperform original-pathways model.

4 Analysis of Expanded Pathways

4.1 Gene Ontology (GO) Term Enrichment Analysis

We performed Gene Ontology (GO) term enrichment analysis for the supplement genes of every pathway, so as to discover some relationships between original pathways and expanded pathways. The results are shown in Figure S3, and the details for the supplement genes of ERK signaling pathway (BRCA), NOTCH signaling pathway (BRCA) and WNT signaling pathway (THCA) are shown in Tables S5, S6 and S7, respectively.

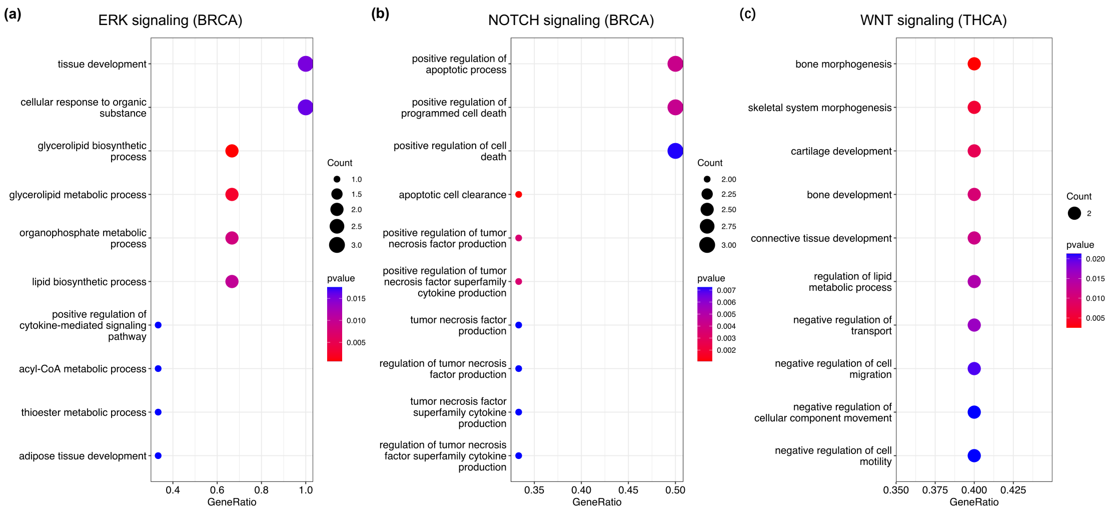

Figure S3: Go Enrichment Analysis

Table S5: GO Enrichment Analysis (ERK signaling - BRCA)

Description	GeneRatio	BgRatio	pvalue	geneID
glycerolipid biosynthetic process	2/3	27/1702	0.000720304	84649/10555
glycerolipid metabolic process	2/3	50/1702	0.002490982	84649/10555
organophosphate metabolic process	2/3	92/1702	0.008369153	84649/10555
lipid biosynthetic process	2/3	101/1702	0.010059627	84649/10555
tissue development	3/3	422/1702	0.015161112	84649/10555/25805
cellular response to organic substance	3/3	428/1702	0.015818615	84649/10555/25805
positive regulation of cytokine-mediated signaling pathway	1/3	10/1702	0.017533207	10555
acyl-CoA metabolic process	1/3	10/1702	0.017533207	84649
thioester metabolic process	1/3	10/1702	0.017533207	84649
adipose tissue development	1/3	10/1702	0.017533207	84649

Table S6: GO Enrichment Analysis (NOTCH signaling - BRCA)

Description	GeneRatio	BgRatio	pvalue	geneID
apoptotic cell clearance	2/6	15/1702	0.001066033	720/7057
positive regulation of tumor necrosis factor production	2/6	27/1702	0.003496794	7057/5005
positive regulation of tumor necrosis factor superfamily cytokine production	2/6	27/1702	0.003496794	7057/5005
positive regulation of apoptotic process	3/6	107/1702	0.004204728	7057/3433/429
positive regulation of programmed cell death	3/6	108/1702	0.004318901	7057/3433/429
positive regulation of cell death	3/6	129/1702	0.007181695	7057/3433/429
tumor necrosis factor production	2/6	39/1702	0.00724334	7057/5005
regulation of tumor necrosis factor production	2/6	39/1702	0.00724334	7057/5005
tumor necrosis factor superfamily cytokine production	2/6	39/1702	0.00724334	7057/5005
regulation of tumor necrosis factor superfamily cytokine production	2/6	39/1702	0.00724334	7057/5005

Table S7: GO Enrichment Analysis (WNT signaling - THCA)

Description	GeneRatio	BgRatio	pvalue	geneID
bone morphogenesis	2/5	14/838	0.00252103	642658/6781
skeletal system morphogenesis	2/5	20/838	0.005187936	642658/6781
cartilage development	2/5	24/838	0.007463464	642658/6781
bone development	2/5	28/838	0.010122866	642658/6781
connective tissue development	2/5	30/838	0.011592773	642658/6781
regulation of lipid metabolic process	2/5	34/838	0.014805605	347/1917
negative regulation of transport	2/5	36/838	0.016545606	347/6781
negative regulation of cell migration	2/5	40/838	0.020285551	347/6781
negative regulation of cellular component movement	2/5	41/838	0.021273649	347/6781
negative regulation of cell motility	2/5	41/838	0.021273649	347/6781

4.2 Single-Gene Survival Analysis

We performed the single-gene survival analysis to validate the significance of the supplement genes in each disease. For one specific gene, we divided the dataset into two groups: high expression group contained the top 50% gene expression level and low expression group contained the others. Then we ploted the Kaplan-Meier curves of the two groups , and identified the most significantly different genes ($p < 0.05$). We displayed the Kaplan-Meier curves in Figure S4. For BRCA, we identified key genes: *LINC01235, TTC36, H2BC4, THBS1, AGPAT2, MMP12.* For THCA, we got *STC1, ND4L, APOD.* For LGG, we obtained *H1-2, LYVE1, MFAP4, PCDHGB6.* These genes were differentially expressed between two groups and might contribute to the performance improvement.

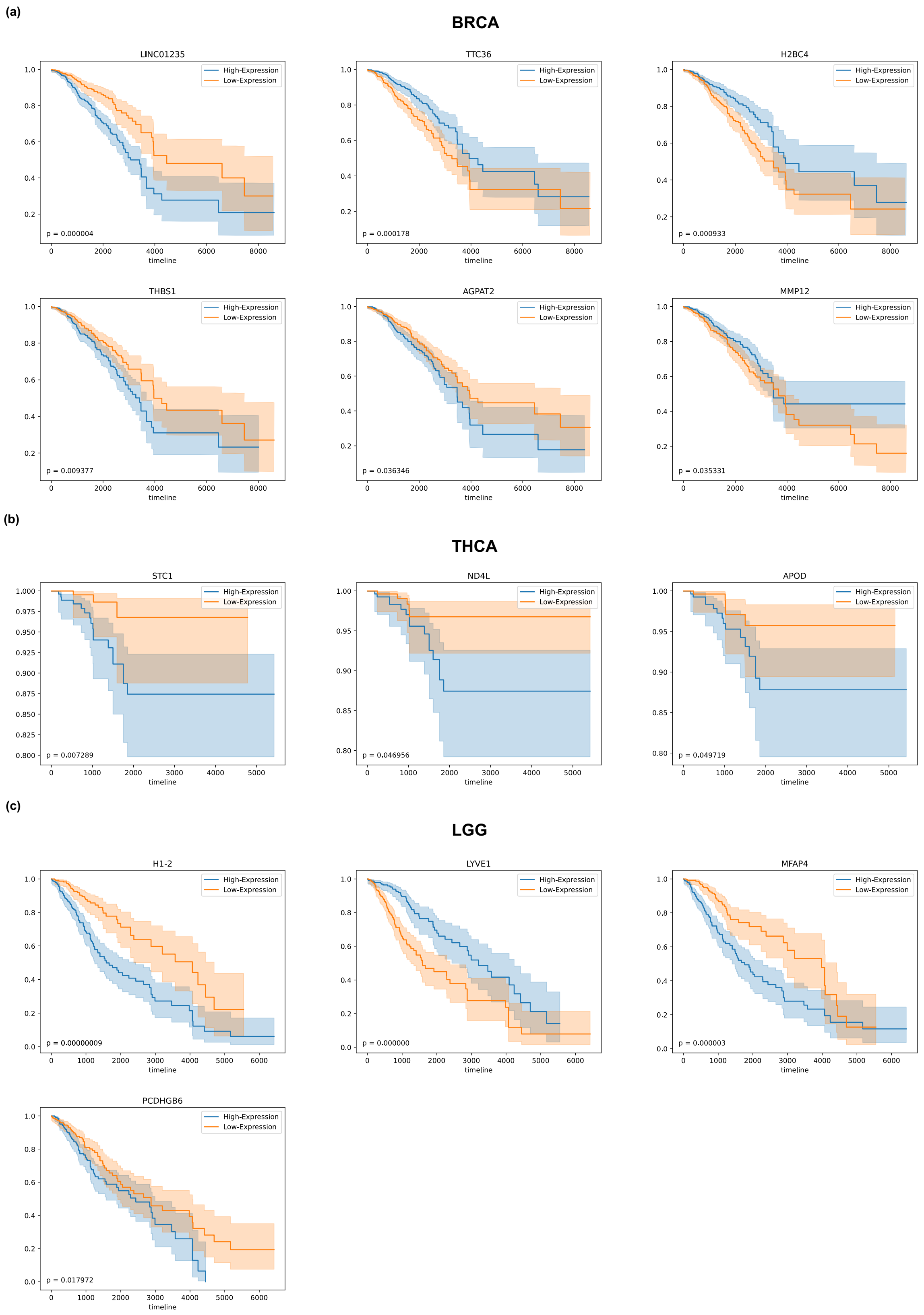

Figure S4: Kaplan Meier curves
